# Congenital Deficiency of Distal Ulna and Dislocation of the Radial Head Treated by Single Bone Forearm Procedure

**DOI:** 10.1155/2014/526719

**Published:** 2014-08-31

**Authors:** Paragjyoti Gogoi, Anshuman Dutta, Arun Kumar Sipani, Arup Kumar Daolagupu

**Affiliations:** ^1^Department of Orthopaedics & Trauma, Silchar Medical College, Silchar, Assam 788014, India; ^2^Department of Orthopaedics & Trauma, Assam Medical College, Dibrugarh, India

## Abstract

Congenital deficiency of part of distal ulna affecting the distal radio-ulnar joint is a rare disorder. It is even rarer to find the association of proximal radio-ulnar joint dislocation along with distal ulnar deficiency. This type of congenital forearm anomaly is difficult to treat. Conversion to a single bone forearm in the expense of pronation-supination movement is a viable option. By doing so the elbow and wrist can be stabilized; however movement is possible in only one plane. We are describing here a girl of 8 years having proximal radio-ulnar joint dislocation along with deficiency of distal ulna treated by converting into a single bone forearm.

## 1. Introduction

Deficiency of ulnar ray in forearm and hand, though rare, is well described [1, 2]. Usually the disorder is seen in hand along with part of the ulna deficient [3]. Ogino et al. have described a classification system where they divided ulnar deficiency into three types such as total absence, partial absence, and hypoplasia of the ulna. The elbow deformities associated are classified into humeroradial synostosis, radial head dislocation, and flexion contracture of the elbow [4]. We are describing here a case of distal ulnar deficiency with expanded tip along with dislocation of the proximal radio-ulnar joint. The forearm has been converted to a single bone as described by Straub (1965) and Lloyd-Roberts (1973) to give stability, but in expense of rotation, that is, pronation-supination movement [5, 6].

## 2. The Case Report

The girl of 8 years at presentation had deformity of the left elbow with a prominence on the outer aspect and weakness of that side in comparison to the normal side. There was no deformity present in the hand. The elbow flexion-extension range of movement was almost normal except about 15-degree restriction of extension. In the forearm supination was restricted considerably; however pronation was normal. The wrist was slightly ulnar deviated and the movements were within normal limits. All the muscle groups were found to have strength less than normal side.

**Figure 1:**
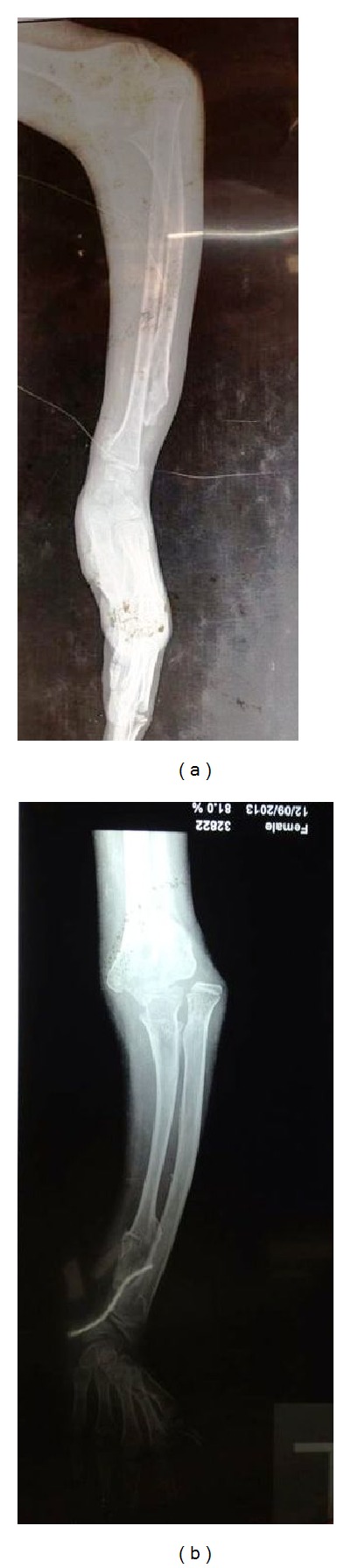
Ulnar deficiency with radial head dislocation.

The X-ray of the forearm showed deficiency of the distal ulna with expanded end and dislocation of the radial head (Figure 1). The humeroulnar joint was normal. The radio-carpel joints were normal and obviously the distal radio-ulnar joint was absent. The carpel bones and the metacarpels were normal.

The decision to convert the deformity into a single bone forearm was taken to give stability and strength to the forearm as well as good cosmesis of the elbow region. It was planned to excise the proximal part of radius and to join the distal part of radius with the proximal part of ulna after osteotomy of the ulna at required level. The distal expanded part of ulna was planned to be left as such so as to support the muscles arising from the part.

**Figure 2:**
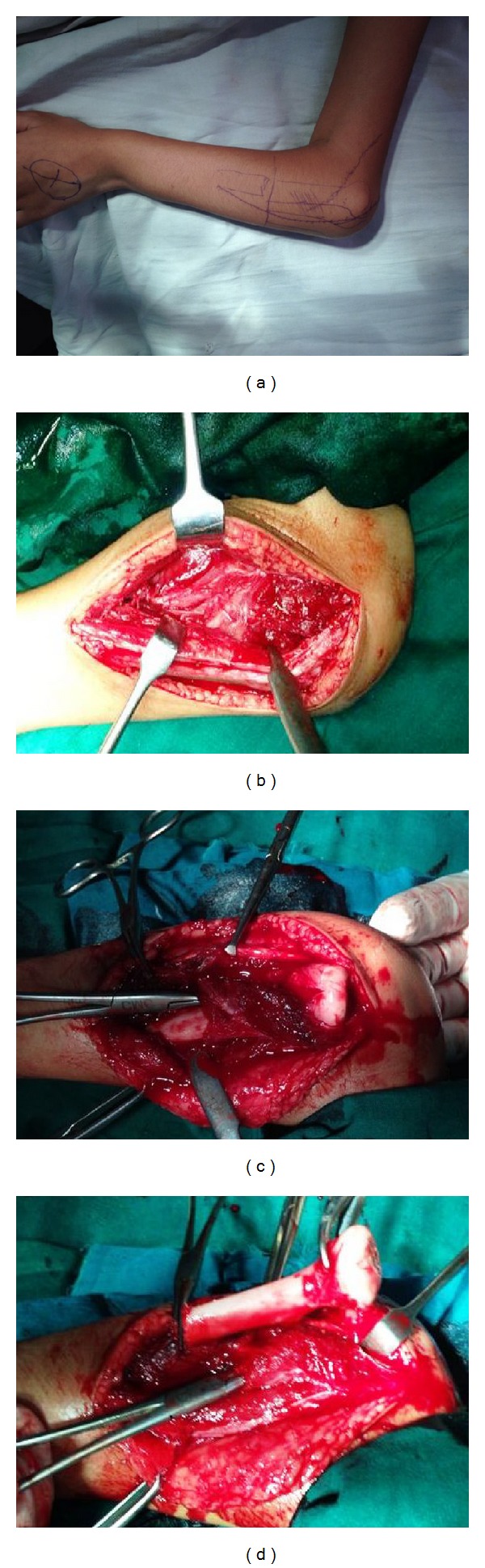
Preoperative pictures.

During the surgery, first the tip of the olecranon and the dislocated radial head were palpated and marked and the possible course of the posterior interosseous nerve was outlined. A gentle curvilinear incision was made starting from the lateral side of the olecranon near the radial head up to the mid forearm. The extensor group of muscles was identified and defined its radial border. They were retracted to expose the supinator muscle and the posterior interosseous nerve was identified. The radial shaft was exposed distal to the nerve pedicle as well as the radial head. The radius was osteotomised and the attached muscles were removed subperiosteally and then the whole proximal radius was removed (Figures 2(a), 2(b), 2(c), and 2(d)).

**Figure 3:**
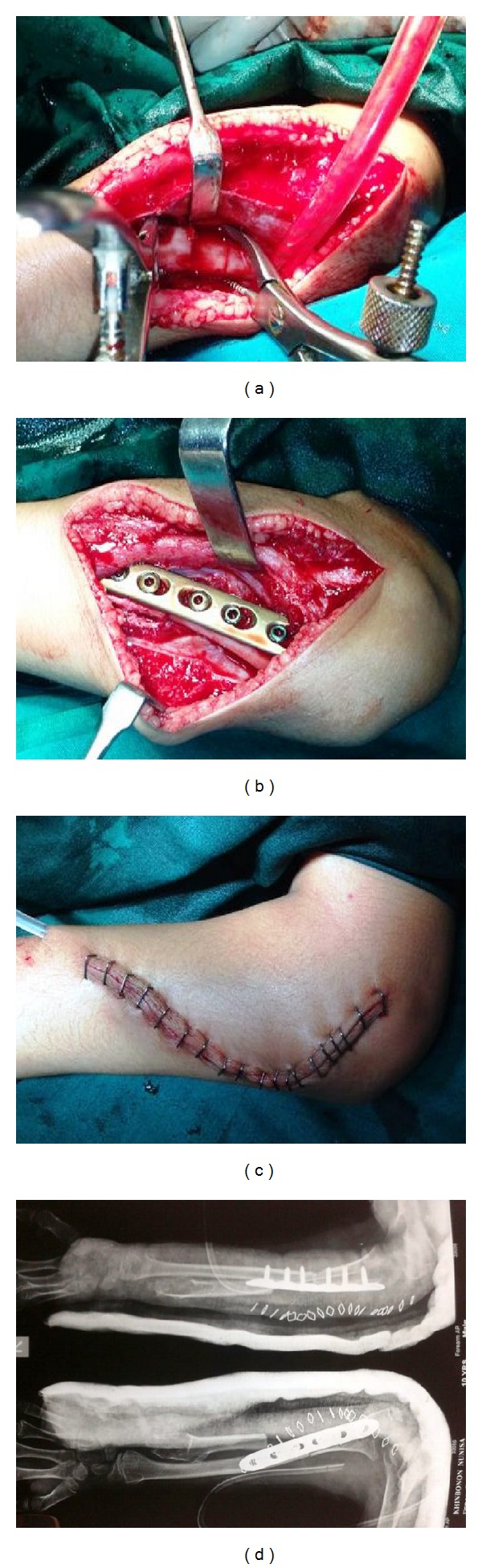
Preoperative pictures.

The ulna was palpated and exposed by a second incision over its periosteum and osteotomised at the same level as that of the radius and then another one centimeter bone was removed from the distal part of ulna. The distal radius was mobilized to come in contact with the proximal osteotomised part of ulna and fixed with a small locking compression plate (Synthes). The surgical wound closed in layers over a suction drain (Figures 3(a), 3(b), 3(c), and 3(d)).

**Figure 4:**
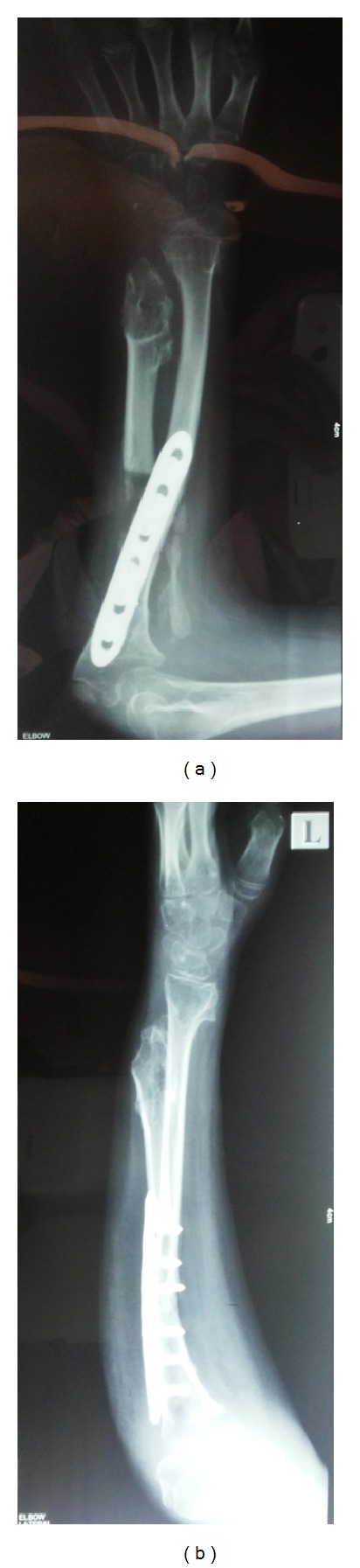
Followup at 3 months. X-ray showing union.

**Figure 5:**
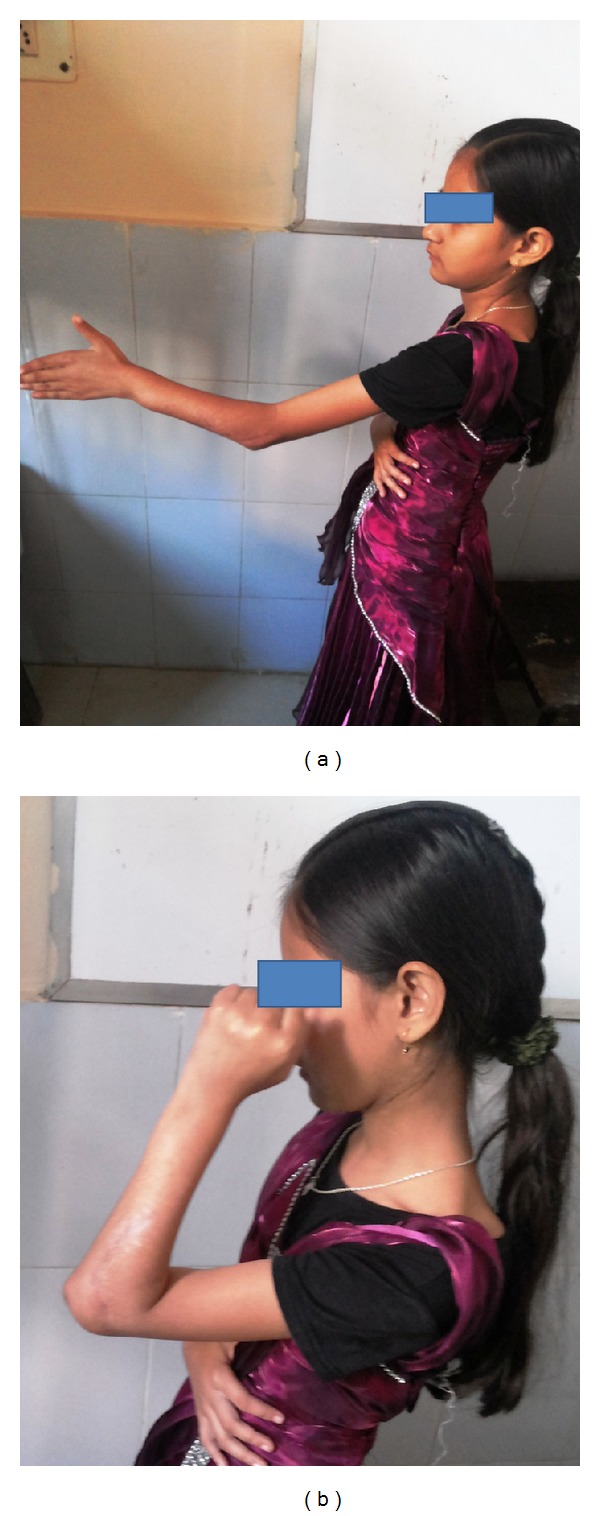
Followup at 3 months. Range of movement.

The patient was followed up regularly and at the end of three months she regained her preoperative muscle strength and had useful range of movement of the elbow and the wrist. Obviously there was no rotational movement of the forearm as expected. The osteotomy site had united and some extra calcification was noted in the excised periosteal sleeve of the radius (Figures 4 and 5).

## 3. Discussion

Ulnar deficiencies are far less common than radial deficiencies. It may have four to ten times less occurrence than its radial counterpart [1, 2]. Also they are not associated with systemic conditions unlike radial deficiencies. However careful physical examination is necessary to rule out other musculoskeletal abnormalities if present.

In congenital deficiency of ulna with radial head dislocation, conversion to a single bone forearm is a viable option. It provides the forearm with the required stability for day to day activities, prevents deformity as well as shortening, and improves hand function. However, the rotational component of the forearm is lost which is to be compensated by the rotation of the arm.

This procedure is well described in the treatment of congenital pseudarthrosis of ulna [7, 8]. In cases of ulnar deficiency where the radial head is not dislocated then the defect can be replaced by free vascularised fibular graft along with reconstruction of the distal radio-ulnar joint [9, 10]. But once the radial head is dislocated and the distal ulnar deficiency is pronounced, then conversion to a single bone forearm is a good alternative. Straub (1965) and Lloyd-Roberts (1973) were credited for description of this technique wherein they osteotomised the radius and the ulna at the same level and then the proximal radius and the distal ulna were resected and finally the proximal ulna was fixed with the distal radius with an intramedullary nail [5, 6, 11].

In our patient we resected the proximal radius but kept the distal ulnar part with a hope of improving the cosmesis and fixed the proximal ulna with distal radius with a small compression plate.

## 4. Conclusion

Congenital ulnar deficiency along with radial head dislocation is a rare condition. If not treated the deformity may progress and there may be bowing and shortening of the forearm. Conversion to single bone forearm gives stability, cosmesis, and better hand function and also it prevents worsening of the deformity.
